# Flammutoxin, a Degradation Product of Transepithelial Electrical Resistance-Decreasing Protein, Induces Reactive Oxygen Species and Apoptosis in HepG2 Cells

**DOI:** 10.3390/foods13010066

**Published:** 2023-12-23

**Authors:** Jianguo Wu, Yu Nong, Bingzhi Chen, Yuji Jiang, Yuanhao Chen, Chuanzheng Wei, Yongxin Tao, Baogui Xie

**Affiliations:** 1 Mycological Research Center, College of Life Sciences, Fujian Agriculture and Forestry University, Fuzhou 350002, China; wjg1419@126.com (J.W.); ny20230601@163.com (Y.N.); calvin18750800131@163.com (Y.C.); weichuanzheng1202@163.com (C.W.); taoyongxin@fafu.edu.cn (Y.T.); 2 College of Food Science, Fujian Agriculture and Forestry University, Fuzhou 350002, China; cbz_2006@163.com (B.C.); jyj1209@163.com (Y.J.); 3 College of Horticulture, Fujian Agriculture and Forestry University, Fuzhou 350002, China

**Keywords:** gastrointestinal cancer, *Flammulina filiformis*, flammutoxin, reactive oxygen species, apoptosis

## Abstract

Proteins from *Flammulina filiformis* were prepared by sodium chloride extraction and fractionated by ammonium sulfate precipitation with increasing saturation degrees to obtain the protein fractions *Ffsp-30*, *Ffsp-50*, *Ffsp-70*, *Ffsp-90*, and *Ffp-90*. Among these protein fractions, *Ffsp-50* possessed the most significant cytotoxic effect against three human gastrointestinal cancer cell lines, viz. HT-29, SGC-7901, and HepG2. SDS-PAGE and MALDI-TOF/TOF MS/MS analyses revealed that flammutoxin (*FTX*) was present as a dominating protein in *Ffsp-50*, which was further evidenced by HPLC-MS/MS determination. Furthermore, native *FTX* was purified from *Ffsp-50* with a molecular weight of 26.78 kDa, exhibiting notable cytotoxicity against gastrointestinal cancer cell lines. Both *Ffsp-50* and *FTX* exposure could enhance intercellular reactive oxygen species (ROS) generation and induce significant apoptosis in HepG2 cells. *FTX* was identified to be relatively conserved in basidiomycetes according to phylogenetic analysis, and its expression was highly upregulated in the primordium as well as the pileus of the fruiting body from the elongation and maturation stages, as compared with that in mycelium. Taken together, *FTX* could remarkably inhibit cell growth and induce ROS and apoptosis in HepG2 cells, potentially participating in the growth and development of the fruiting body. These findings from our investigation provided insight into the antigastrointestinal cancer activity of *FTX*, which could serve as a biological source of health-promoting and biomedical applications.

## 1. Introduction

Gastrointestinal (GI) cancers, occurring in the colon, stomach, and liver, are considered as the most diagnosed and lethal cancers in the world. The unpleasant outcomes of traditional therapeutic approaches highlight the ever-growing need to explore novel and effective natural anticancer agents [1]. Recently, mushroom-originated proteins have received growing scientific attention as a promising candidate for anticancer agents which could exert their bioactive/pharmacological effects through different mechanisms of action, exemplified by DNase activities, endoplasmic reticulum stresses, and ubiquitin-mediated pathways [2].

*Flammulina filiformis* (*F. filiformis,* known as *Flammulina velutipes* previously), belonging to the class Agaricomycetes and the phylum Basidiomycota, is a popular commercial edible mushroom in Asia which is generally considered a low-calorie delicacy possessing important nutritional and medicinal values [3]. Phytochemical and pharmacological studies have shown that the phenolic acids, flavonoids, sesquiterpenes, polysaccharides, proteins, and glycoproteins present in *F. filiformis* display a wide range of biological/pharmacological activities, including antioxidant, antibacterial, anti-inflammatory, antitumor, antihypertensive, antiatherosclerotic, thrombosis-inhibiting, cholesterol-lowering, intestinal-flora-regulating, memory-enhancing, neuroprotective, hepatoprotective, and immunomodulatory properties [4,5,6]. Recently, biological molecules like polysaccharides, proteins, and glycoproteins in *F. filiformis* have attracted the attention of researchers owing to their beneficial health effects. Many studies have focused on the extraction, purification, structural characteristics, and bioactivities of *F. filiformis* polysaccharides [7]. However, much less is known about the proteins of *F. filiformis*, mainly their pharmacological benefits or bioactive functions.

Hitherto, several functional proteins have been isolated from the fruiting body of *F. filiformis*, including the ribosome-inactivating proteins velutin, velin, flammin, and flammulin [8,9,10], the fungal immunomodulatory protein FIP-*fve* [11], the acidic glycoprotein proflamin [12], and the pore-forming cytolysin flammutoxin (*FTX*) [13]. FIP-*fve* and proflamin have been demonstrated to exhibit immunomodulatory and anticancer properties [12,14]. *FTX*, first isolated from the fruiting body of *F. filiformis*, has been found to cause swelling and inhibit the respiration of Ehrlich ascites tumor cells [15,16]. Furthermore, it was reported that the protein *Zb* sharing the exact same N-terminal amino acid (AA) sequence of PQVKTSWEDLANLGWPIQQV with *FTX* was found to significantly inhibit human gastric adenocarcinoma cell line MGC80-3 cells with an IC_50_ value of 0.75 μg/mL [17]. These encouraging findings prompted us to examine the potential growth-inhibitory and apoptosis-inducing effects of *FTX* against GI cancer cell lines.

In our present work, different protein fractions (*Ffsp-30*, *Ffsp-50*, *Ffsp*-*70*, *Ffsp*-*90*, and *Ffp*-*90*) from the fruiting body of *F. filiformis* were prepared by sodium chloride (NaCl) extraction and ammonium sulfate (NH_4_)_2_SO_4_ precipitation. Native *FTX* was further purified from *Ffsp*-*50*, which was analyzed by sodium dodecyl sulfate polyacrylamide gel electrophoresis (SDS-PAGE) and high-performance gel permeation chromatography (HPGPC). The molecular weight (*M_w_*) and AA sequence were further determined by MALDI-TOF MS/MS. The growth-inhibitory effects of the protein fractions and purified *FTX* on the HT-29, SGC-7901, and HepG2 cell lines were evaluated by standard 3-(4, 5-dimethylthiazolyl-2)-2,5-diphenyltetra-zolium bromide (MTT) assay. The apoptosis-inducing effect of *Ffsp*-*50* and *FTX* on HepG2 cells was confirmed by DAPI nuclear staining and flow cytometry analysis. Intracellular ROS level was determined by 2′,7′-dichlorodihydrofluorescein diacetate (DCFH-DA) staining. Moreover, phylogenetic analysis was performed to assess the evolutionary relationship of *FTX* from *F. filiformis* with other fungal *FTX* homologue proteins. And the *FTX* gene expression profile during the growth and development of *F. filiformis* was analyzed by real-time quantitative PCR (qPCR).

## 2. Materials and Methods

### 2.1. Chemicals and Materials

Different developmental tissue samples of dikaryon strain C18 of *F. filiformis* were provided by Fujian WanChen Biotechnology Group Co., Ltd. (Zhangzhou City, China), including the mycelium, primordium, young fruiting body, as well as the fruiting body (stipe and pileus) in the elongation and mature stages. The strain was deposited in the Fujian edible fungi germplasm resource collection center (Accession Number: F0421). Three human GI cancer cell lines, i.e., HT-29, SGC-7901, and HepG2, were obtained from the Cell Bank of Type Culture Collection of the Chinese Academy of Sciences (Shanghai, China). 3-(4,5-dimethylthiazolyl-2)-2,5-diphenyltetra-zolium bromide (MTT) and 4’, 6-diamidino-2-phenylindole dihydrochloride (DAPI) were purchased from Sigma-Aldrich (St. Louis, MO, USA). Fetal bovine serum (FBS), trypsin, and penicillin–streptomycin were obtained from Hyclone Laboratories, Inc. (Logan, UT, USA). Dulbecco’s modified Eagle medium (DMEM) and RPMI1640 medium were from Gibco (Grand Island, NY, USA). McCoy’s 5A (GM3109-500ML) medium was purchased from Gen-View Scientific, Inc. (El Monte, FL, USA). The BCA protein assay kit was obtained from Beyotime Biotechnology (Shanghai, China). Annexin V-fluorescein isothiocyanate-(FITC)/propidiumiodide (PI) apoptosis detection kit and 2′, 7′-dichlorodihydrofluorescein diacetate (DCFH-DA) were obtained from Nanjing Key GenBiotech Co., Ltd. (Nanjing, China). E.Z.N.A.^®^ Plant RNA Kit was purchased from OMEGA Biotechnology Co., Ltd. (Stamford, CT, USA). TransScript^®^ All-in-One First-Strand cDNA Synthesis SuperMix for qPCR (One-Step gDNA Removal) Kit and PerfectStart™ Green qPCR SuperMix were from TransGen Biotech (Beijing, China). All chemical reagents, including paraformaldehyde (PFA), NaCl, (NH_4_)_2_SO_4_, Tris-HCl, etc. were of analytical grade and supplied by Solarbio Biotechnology (Beijing, China).

### 2.2. Preparation and Analysis of Protein Fractions

Different protein fractions from the fruiting bodies of *F. filiformis* were obtained by NaCl extraction and (NH_4_)_2_SO_4_ precipitation [18]. In brief, the fruiting bodies of *F. filiformis* (2 kg) in the elongation stage were cut to pieces and lyophilized, ground, and soaked overnight in chilled 0.15 M NaCl (20 mL/g) at 4 °C, which was then centrifuged at 8000× *g* for 15 min to obtain the supernatant. The different protein fractions (*Ffsp*-*30*, *Ffsp*-*50*, *Ffsp*-*70*, *Ffsp*-*90*, and *Ffp*-*90*) were successively precipitated by the stepwise addition of solid (NH_4_)_2_SO_4_ with increasing saturation degrees (0–30%, 30–50%, 50–70%, 70–90%, and 0–90%). All these proteins were desalted by membrane dialysis (*M_w_* cutoff 7.0 kDa) against deionized water and lyophilized. Protein content was determined by the bicinchoninic acid (BCA) method using bovine serum albumin (BSA) as the reference standard.

*M_w_* distributions of protein fractions were further analyzed using SDS-PAGE (12% *w*/*v*). Dominant protein bands were subsequently analyzed with a RapiFlex MALDI-TOF/TOF MS/MS (Bruker Daltonik GmbH, Bremen, Germany). Moreover, the protein fraction *Ffsp*-*50* was determined using an UltiMate 3000 RSLCnano HPLC system (Thermo Scientific, Waltham, MA, USA) coupled to a Q Exactive Plus Hybrid Quadrupole Orbitrap Mass Spectrometer (Thermo Scientific) by Sangon Biotech (Shanghai) Co., Ltd. The obtained sequences were then searched by local BLAST searches against a protein database of *F. filiformis* provided by the Mycological Research Center of Fujian Agriculture and Forestry University. The resulting protein was predicted for the structural and functional domain using the Conserved Domain Search service (CD-Search).

### 2.3. Purification, Analysis, and Identification of Native FTX Protein

Briefly, *Ffsp-50* was dissolved in 0.02 M Tris-HCl buffer solution (pH 8.0), which was centrifuged at 12,000× *g* for 10 min at 4 °C and filtered with 0.45 µm pore-sized filters to remove insoluble proteins. The resulting filtrate was applied to Cellufine Q-500 column (JNC Corp., Tokyo, Japan) pre-equilibrated with 0.02 M Tris-HCl buffer solution (pH 8.0), which was then eluted with a stepwise elution of 0, 0.1 and 0.2 M NaCl in the buffer. The eluents were combined by measuring absorption at 280 nm. They were dialyzed (*M_w_* cutoff 8~10 kDa) against deionized water and further lyophilized. 

The purity of the obtained protein was analyzed by SDS-PAGE (12% *w*/*v*) and high-performance gel permeation chromatography (HPGPC). HPGPC was performed using an EasySep-3030 HPLC system (Unimicro Technologies, Shanghai, China), with a TSK-Gel G2000SWxL column (7.8 mm I.D. × 30 cm, 5 μm; Tosoh Biosciences, Tokyo, Japan) preceded by a TSK-Gel G2000SW guard column (6.0 mm I.D. × 4 cm, 7 μm; Tosoh Biosciences, Tokyo, Japan). The chromatographic conditions were as follows: column temperature 25 °C; 30 mM Tris-acetic acid (HAC) buffer (pH 6.0) as mobile phase; flow rate at 0.5 mL/min; ultraviolet detection at 280 nm. Furthermore, the AA sequence and *M_w_* of the purified protein were analyzed with a RapiFlex MALDI-TOF/TOF MS/MS by Sangon Biotech (Shanghai, China) Co., Ltd. The resulting AA sequences of peptides from the protein were submitted for BLAST searches against the protein database of *F. filiformis* provided by the Mycological Research Center of Fujian Agriculture and Forestry University.

### 2.4. MTT Assay

HT-29, SGC-7901, and HepG2 cells were cultured in a medium containing 10% FBS, 100 units/mL penicillin and 100 μg/mL streptomycin in a 37 °C humidified incubator with 5% CO_2_ atmosphere. The growth-inhibitory effect of different protein fractions (*Ffsp-30*, *Ffsp-50*, *Ffsp*-*70*, *Ffsp*-*90*, and *Ffp*-*90*) and *FTX* was tested using the standard MTT assay [19]. Briefly, cells with a density of 1 × 10^5^ cells/mL were plated in and permitted to adhere to 96-well plates for 24 h. The protein fractions and *FTX* were diluted with the medium and filtered using 0.22 µm syringe filters. The cells were then exposed to the indicated concentrations of protein fractions (24 h) and *FTX* (24, 48, and 72 h). Cells that received the same volume of medium were used as control. After incubation, 20 μL of MTT reagent (5 mg/mL in PBS) was added and cells were incubated for another 4 h. The formazan produced by the viable cells was solubilized by the addition of 100 μL dimerthyl sulfoxide. The suspension was placed on a microvibrator for 10–15 min and absorbance was recorded at 570 nm using a multifunctional enzyme-labeled instrument (TECAN Infinite M200 PRO, Männedorf, Switzerland). Cell viability was expressed as a percentage relative to the vehicle-treated controls.

### 2.5. DAPI Staining

HepG2 cells in the logarithmic growth phase were planted into 6-well flat-bottomed plates with a density of 1 × 10^5^ cells/mL in 2 mL of culture medium and incubated for 24 h at 37 °C in a 5% CO_2_ atmosphere. The cells were exposed to various concentrations of *Ffsp-50* (0, 25, 50, and 100 µg/mL) and *FTX* (0, 12.5, 25, and 50 µg/mL) for another 24 h after washing twice with PBS. The cells were fixed with 4% PFA in PBS for 10 min at room temperature, then washed twice with PBS and stained with 2 µg/mL of DAPI for 10 min at room temperature. The stained cells were visualized using an inverted fluorescence microscope under ×200 magnification (Leica DMi8, Leica Microsystems, Wetzlar, Germany).

### 2.6. Intracellular ROS Level Determination

Briefly, HepG2 cells at a density of 1 × 10^5^ cells/mL were seeded on 6-well bottom plates and allowed to attach for 24 h. They were then grouped and challenged with different concentrations of *Ffsp-50* (0, 25, 50, and 100 µg/mL) and *FTX* (0, 12.5, 25, and 50 µg/mL). The cells were washed with PBS 3 times and stained with 10 μmol/L DCFH-DA at 37 °C for 30 min in the dark according to the manufacturer’s instructions. Intracellular ROS generation was visualized using an inverted fluorescence microscope under ×200 magnification at an excitation wavelength of 485 nm and emission wavelength of 528 nm.

### 2.7. Analysis of Apoptosis by Flow Cytometer

The apoptosis-eliciting effect of *Ffsp-50* and *FTX* on HepG2 cells was determined with Annexin V-FITC/PI double staining according to the manufacturer’s instructions. After incubation with different concentrations of *Ffsp-50* (0, 25, 50, and 100 µg/mL) and *FTX* (0, 12.5, 25, and 50 µg/mL) for 24 h, the apoptosis of HepG2 cells was analyzed immediately using a flow cytometer (FACS Calibur, Agilent NovoCyte Penteon, Lexington, MA, USA). The percentage of early apoptotic cells was calculated by Annexin V^+^/PI^−^ staining, while the percentage of late apoptotic cells was calculated by Annexin V^+^/PI^+^ staining.

### 2.8. Phylogenetic Tree Construction

To generate the phylogenetic tree, the AA sequence of *FTX* (Sequence ID: BAA76510.1) was searched using protein–protein BLAST in NCBI. The homologous proteins of *FTX* from representative fungi were employed for phylogenetic analysis using the MEGA 5.0 software program [20] after a multiple alignment of data using ClustalX [21]. Distances were calculated using distance options according to the Kimura two-parameter model [22]. Cluster analyses were performed using the neighbor-joining algorithm [23]. Tree topology was evaluated by bootstrap analysis with 1000 replications [24].

### 2.9. RNA Isolation and qPCR

Total RNA was isolated from different developmental tissue samples using an E.Z.N.A.^®^ Plant RNA Kit according to the manufacturer’s protocol. Extracted RNA was quantified using a NanoND-1000 spectrophotometer (NanoDrop Technologies, Wilmington, DE, USA). The first strand of cDNA was generated using the TransScript^®^ All-in-One First-Strand cDNA Synthesis SuperMix for qPCR (One-Step gDNA Removal) Kit. All cDNA was stored at −20 °C for subsequent experiments. Subsequently, qPCR was executed using PerfectStart™ Green qPCR SuperMix and a CFX96^TM^ Real-Time PCR detection system (Bio-Rad Laboratories, Hercules, CA, USA). A 20 μL reaction system was prepared according to the manufacturer’s instructions consisting of 2 µL of cDNA, 0.4 µL each of forward and reverse primers for the *FTX* gene (forward 5′-TCAATGGTGGTGCTACAACAG-3′ and reverse 5′-GGGAGGCGTGGTTAGTGATG-3′), 10 µL of qPCR supermix, and 7.2 µL of ddH_2_O. qPCR amplification of the *FTX* gene was conducted under the following conditions: 95 °C for 2.5 min, followed by 40 cycles of 94 °C for 5 s, 58 °C for 30 s, and 60 °C for 5 s. Glyceraldehyde-3-phosphate dehydrogenase (GAPDH) and β-actin (ACTB)-encoding genes were employed as reference genes for qPCR data normalization [25]. Amplifications for each sample were performed with three technical replicates. Relative gene expression levels were calculated using the 2^−ΔΔCt^ method [26].

### 2.10. Statistical Analysis

All experiments were carried out with three independent replicates. The resulting data were represented as means ± standard deviation ($\bar{x}$ ± SD) from triplicate samples. Statistical comparison was conducted by one-way analysis of variance (ANOVA) using SPSS software version 22.0 (SPSS, Chicago, IL, USA). *p* values less than 0.05 were considered statistically significant.

## 3. Results

### 3.1. Preparation and Analysis of Protein Fractions

**Figure 1:**
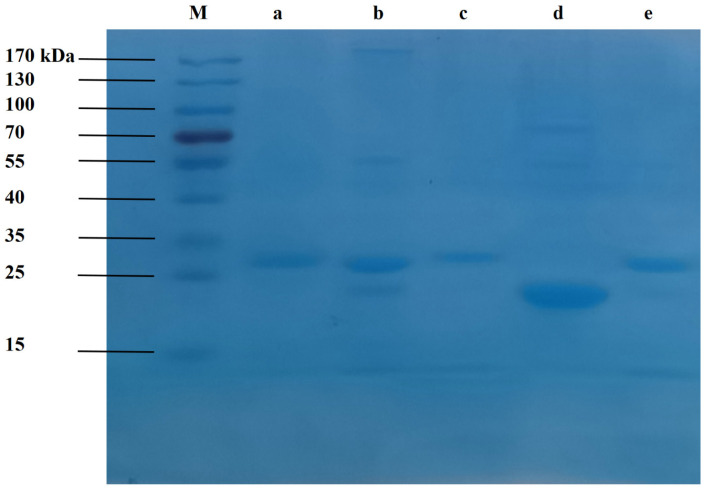
SDS-PAGE analysis of protein fractions from the fruiting bodies of *F. filiformis*. Samples were electrophoresed on 12% gel and stained with Coomassie brilliant blue. Column M represents a pre-stained protein marker with *M_w_* range of 15–170 kDa; columns a, b, c, d, and e represent *Ffsp-30*, *Ffsp-50*, *Ffsp-70*, *Ffsp-90*, and *Ffp-90*, respectively.

Proteins from *F. filiformis* were systematically partitioned and evaluated for cytotoxicity against GI cancer cell lines. Different protein fractions, viz. *Ffsp-30*, *Ffsp-50*, *Ffsp*-*70*, *Ffsp*-*90*, and *Ffp*-*90*, were achieved with the yield of 0.072%, 1.32%, 0.485%, 0.0096%, and 1.62% with a total protein content of 33.07%, 40.71%, 36.57%, 33.75%, and 31.36%, respectively. This finding indicated that *Ffsp-50* was obtained with a relatively higher yield and total protein content than the other protein fractions. SDS-PAGE analysis showed that *Ffsp-30, Ffsp-50, Ffsp*-*70,* and *Ffp-90* produced a strong staining protein band with a molecular weight (*M_w_*) of 25~35 kDa, as shown in Figure 1 column a, b, c, and e, respectively. Moreover, *Ffsp-50* seemed to possess more abundant protein components than other protein fractions. Nevertheless, a high-staining-intensity protein band with an *M_w_* approaching 25 kDa was observed in *Ffsp-90*, as seen in Figure 1 column d. Further, MALDI-TOF/TOF MS/MS determination revealed that the dominant protein band in *Ffsp-30*, *Ffsp-50*, *Ffsp-70*, and *Ffp-90* was confirmed as *FTX* (25~35 kDa, *M_w_* 30.095 kDa, GenBank accession no. BAA76510.1), as well as *FDS* protein (15~25 kDa, *M_w_* 22.809 kDa, GenBank accession no. ACZ59468.1) in *Ffsp-90*. HPLC-MS/MS was further performed to determine the protein compositions of *Ffsp-50*. Table 1 shows transepithelial electrical resistance (TEER)-decreasing protein (TDP, GenBank accession no. BAA76510.1) was one of the main proteins in *Ffsp-50*. TDP was the precursor of or identical to *FTX* [27], which could produce multiple *FTX* family proteins by partial C-terminal truncation [28]. Therefore, these findings suggested that *FTX* was the dominating protein in the different protein fractions except for *Ffsp-90*.

### 3.2. FTX Protein Purification and Characterization

**Figure 2:**
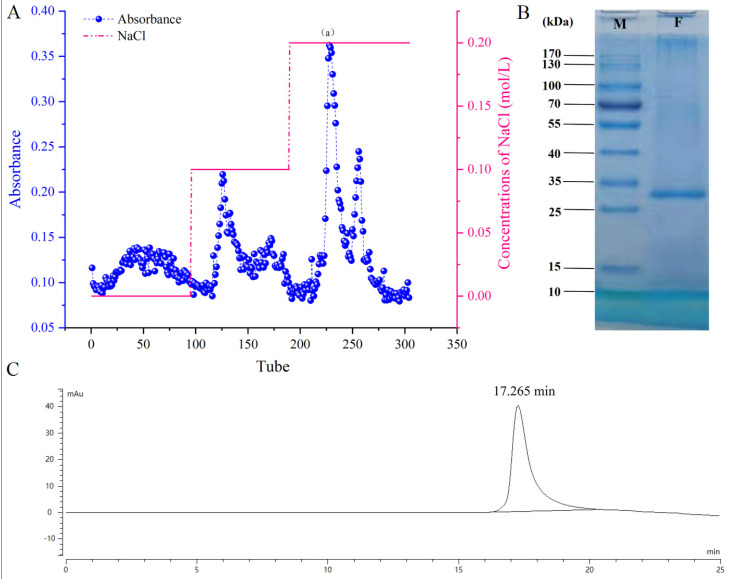
Purification and analysis of *FTX* protein. (**A**) Purification of native *FTX* from *Ffsp-50* on a Cellufine Q-500 strong anion-exchange column. *FTX* was obtained from section (a) of eluents of 0.2 M NaCl in 0.02 M Tris-HCl buffer solution (pH 8.0). (**B**) SDS-PAGE analysis of purified *FTX* from the fruiting body of *F. filiformis*. Column M represents the pre-stained protein marker with *M_w_* range of 10–170 kDa; column F represents purified *FTX*. (**C**) Chromatogram of purified *FTX* determined by HPGPC method.

**Figure 3:**
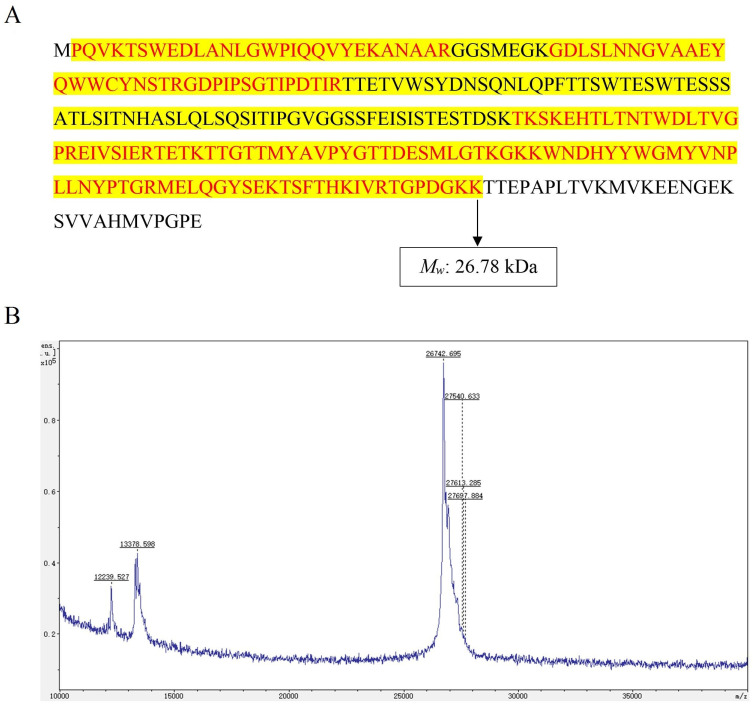
AA sequence and molecular weight of *FTX* determined by MALDI-TOF/TOF MS/MS. (**A**) The deduced AA sequence of *FTX* precursor (GenBank accession no. BAA76510.1). The red parts indicate the peptide fragments (sequence coverage: 62%) detected by MALDI-TOF/TOF MS/MS; the region highlighted in luminous yellow denotes the presumed AA sequence of purified *FTX* lacking the initial Met (M) and C-terminal 30 AA residues (241 AA residues, *M_w_* 26.78 kDa). (**B**) *FTX* molecular weight detected by MALDI-TOF/TOF MS/MS.

Since *FTX* was an essential protein in different protein fractions, it was reasonable to purify *FTX* for further investigation. Owing to its high yield and total protein content, *Ffsp-50* was subsequently subjected to a Cellufine Q-500 column. After gradient elution, *FTX* was obtained from section (a) of eluents of 0.2 M NaCl in buffer solution, as shown in Figure 2A. SDS-PAGE and HPGPC analyses were further performed to characterize the purity of protein, resulting in a single protein band stained by Coomassie brilliant blue (*M_w_* 25~35 kDa, as shown in Figure 2B) and a signal peak (retention time: 17.265 min, as seen in Figure 2C). According to previous reports [28,29], the *FTX* precursor known as TDP consists of 272 AA residues with an *M_w_* of approximately 31 kDa, the complete AA sequence of which was listed in Figure 3A (https://www.ncbi.nlm.nih.gov/protein/BAA76510.1, accessed on 26 September 2022). As detected by MALDI-TOF/TOF MS/MS, the purified protein was identified as *FTX* based on the AA sequence of peptide fragments, with 63% coverage as marked in red in Figure 3A. Moreover, the *M_w_* of purified *FTX* was measured as 26.74 kDa (see in Figure 3B), thus suggesting that it is the degradation product of *FTX* precursor, namely mature *FTX* after modification. Purified *FTX* was consequently presumed to contain 241 AA residues with an *M_w_* of 26.78 kDa, ranging from the 2nd AA residue P to the 242nd AA residue K, which lacked the initial Met (M) and the C-terminal 30 residues (see the luminous yellow region in Figure 3A) [13].

### 3.3. Evaluation of Cell-Growth-Inhibitory Activity

**Figure 4:**
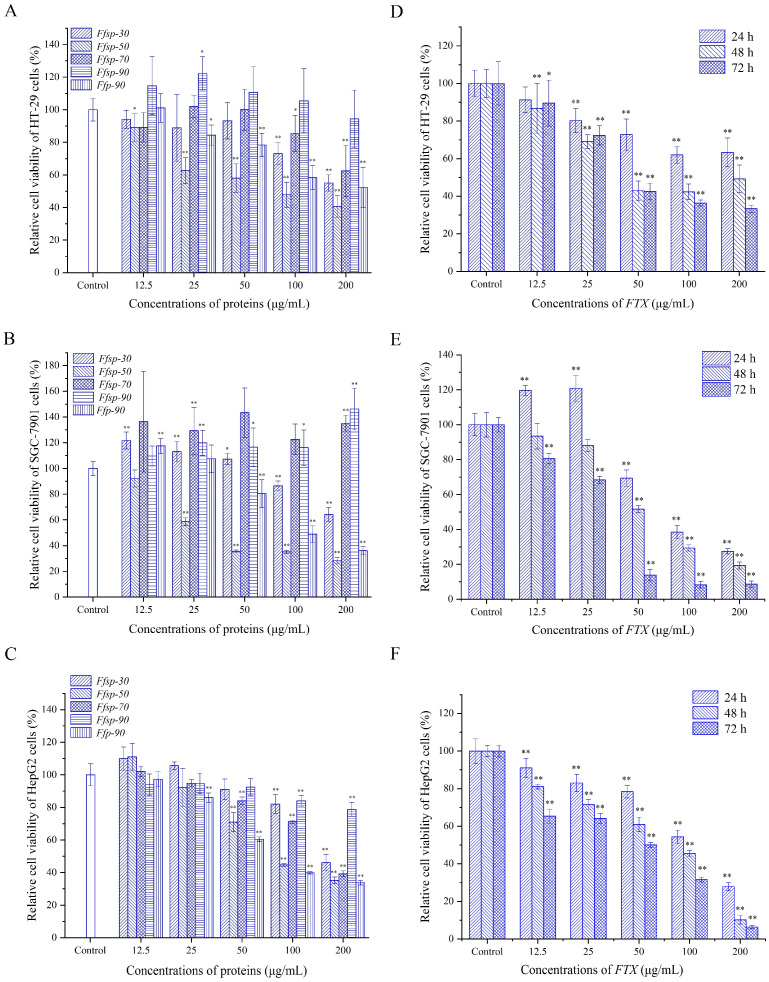
Growth-inhibiting effects on three human gastrointestinal cancer cell lines. Using a standard MTT assay, the relative cell viabilities of HT-29, SGC-7901, and HepG2 cells were determined after treatment with indicated concentrations of (**A**–**C**) *Ffsp-30*, *Ffsp-50*, *Ffsp-70*, *Ffsp-90*, and *Ffp-90* for 24 h or (**D**–**F**) *FTX* for 24, 48, and 72 h. Values are represented as the percentage of viable cells, with vehicle-treated cells regarded as 100% viable. Data are presented as mean ± S.D. of three independent experiments. * *p* < 0.05, ** *p* < 0.01 compared with vehicle-treated controls.

A standard MTT assay was conducted to evaluate the growth-inhibitory activities of the protein fractions (viz. *Ffsp-30*, *Ffsp-50*, *Ffsp-70*, *Ffsp-90*, and *Ffp-90*) and *FTX* against three human GI cancer cell lines, viz. HT-29, SGC-7901, and HepG2. As presented in Figure 4A–C, the protein fractions decreased GI cancer cell viability by different degrees. Particularly, *Ffsp-50* exhibited a conspicuous growth-inhibitory effect against HT-29, SGC-7901, and HepG2 cells with half maximal inhibitory concentration (IC_50_) values of 92.40, 60.75, and 102.20 μg/mL, respectively. Nevertheless, *Ffsp-90* displayed no cytotoxicity toward HT-29 and SGC-7901 cells, and lowly cytotoxic to HepG2 cells, probably due to the absence or low concentration of *FTX*. Overall, these protein fractions exerted a more substantial cell-inhibitory effect against HepG2 cells than the other two cancer cell lines, showing a dose-dependent relationship.

*FTX*, purified from effective anticancer protein fraction *Ffsp-50*, was further investigated for the growth-inhibitory effect against HT-29, SGC-7901, and HepG2 cells. As shown in Figure 4D–F, *FTX* evidently restrained the growth of HT-29 cells, with IC_50_ values of 295.37, 89.22, and 67.48 μg/mL at 24, 48, and 72 h, respectively. Nevertheless, no noticeable cytotoxicity was observed when SGC-7901 cells were exposed to 12.5 and 25 μg/mL of *FTX* at 24 and 48 h. *FTX* prominently decreased the cell viability of SGC-7901 cells with an IC_50_ value of 28.62 μg/mL at 72 h (*p* < 0.01). Furthermore, *FTX* outstandingly suppressed the growth of HepG2 cells in a time- and dose-dependent manner (*p* < 0.01); which IC_50_ values at 24, 48, and 72 h were 107.77, 53.96, and 36.8 μg/mL, respectively. Based on the above findings, HepG2 cells were further employed to investigate ROS generation and apoptosis induction.

### 3.4. Apoptosis-Inducing Activity

**Figure 5:**
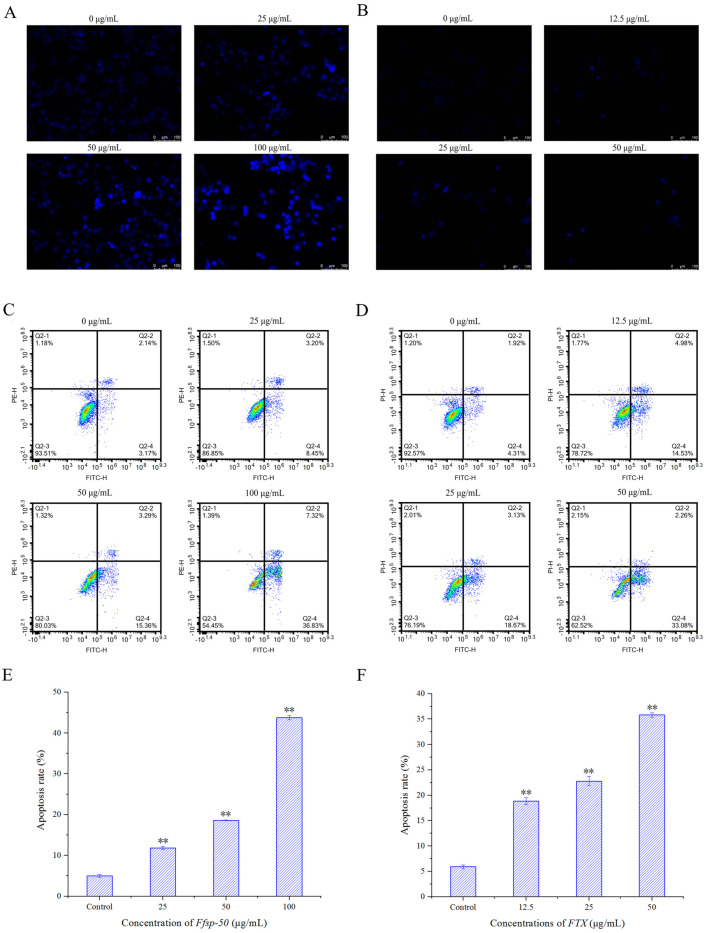
Morphological changes in HepG2 cells examined by DAPI nuclear staining under a fluorescence microscope after challenge with indicated concentrations of (**A**) *Ffsp-50* (0, 25, 50, and 100 μg/mL) and (**B**) *FTX* (0, 12.5, 25, and 50 μg/mL) for 24 h. Images are representative of three independent experiments (magnification ×200). Apoptosis-inducing effects of *Ffsp-50* (**C**,**E**) and *FTX* (**D**,**F**) in HepG2 cells. HepG2 cells were treated with indicated concentrations of *Ffsp-50* (0, 25, 50, and 100 μg/mL) and *FTX* (0, 12.5, 25, and 50 μg/mL) for 24 h and analyzed by flow cytometry with Annexin V/PI double staining. Representative FACS analysis scattergrams of Annexin V/PI show the four different cell populations, which are described as follows: double-negative stained cells (Annexin V^−^/PI^−^, lower left) indicating the live cell population; Annexin V positive/PI negative stained cells (Annexin V^+^/PI^−^, lower right) and double−positive (Annexin V^+^/PI^+^, upper right) stained cells showing early and late apoptotic cells, respectively; Annexin V negative/PI positive stained cells (Annexin V^−^/PI^+^, upper left) denoting dead cells. Data shown are presented as mean ± SD of three independent experiments. ** *p* < 0.01 compared with vehicle−treated controls.

In order to determine whether the growth-inhibitory effects of *Ffsp-50* and *FTX* on HepG2 cells were partially attributable to their apoptosis-triggering potential, morphological alterations were visualized by DAPI nuclear staining under a fluorescence microscope after treatment with different concentrations of *Ffsp-50* (0, 25, 50, and 100 μg/mL) and *FTX* (0, 12.5, 25, and 50 μg/mL) for 24 h. Exposure to both *Ffsp-50* and *FTX* led to a decreased number of HepG2 cells along with several visible apoptotic morphological changes, including increased brightness, cell shrinking, nuclear deformation, and chromatin condensation (pyknosis), as shown in Figure 5A,B, respectively. Flow cytometry analysis was further performed to quantify the extent of apoptosis in HepG2 cells with Annexin V/PI double staining. In Figure 5C,D, the lower right (Annexin V^+^/PI^−^) and upper right (Annexin V^+^/PI^+^) indicate early and late apoptotic cells, respectively, in representative fluorescence-activated cell sorting (FACS) analysis scattergrams. The percentage of apoptotic cells following challenge with 0, 25, 50, and 100 μg/mL of *Ffsp-50* was 4.89%, 11.86%, 18.61%, and 43.73%, respectively. Moreover, the percentage of apoptotic cells after treatment with 0, 12.5, 25, and 50 μg/mL of *FTX* was 5.89%, 18.88%, 22.78%, and 35.80% respectively. The number of apoptotic cells, particularly early apoptotic cells, was dramatically increased by both *Ffsp-50* and *FTX* (*p* < 0.01), as seen in Figure 5E,F, respectively. Our finding demonstrated significant apoptosis-triggering activities elicited by *Ffsp-50* and *FTX*. 

### 3.5. Intracellular ROS Generation

**Figure 6:**
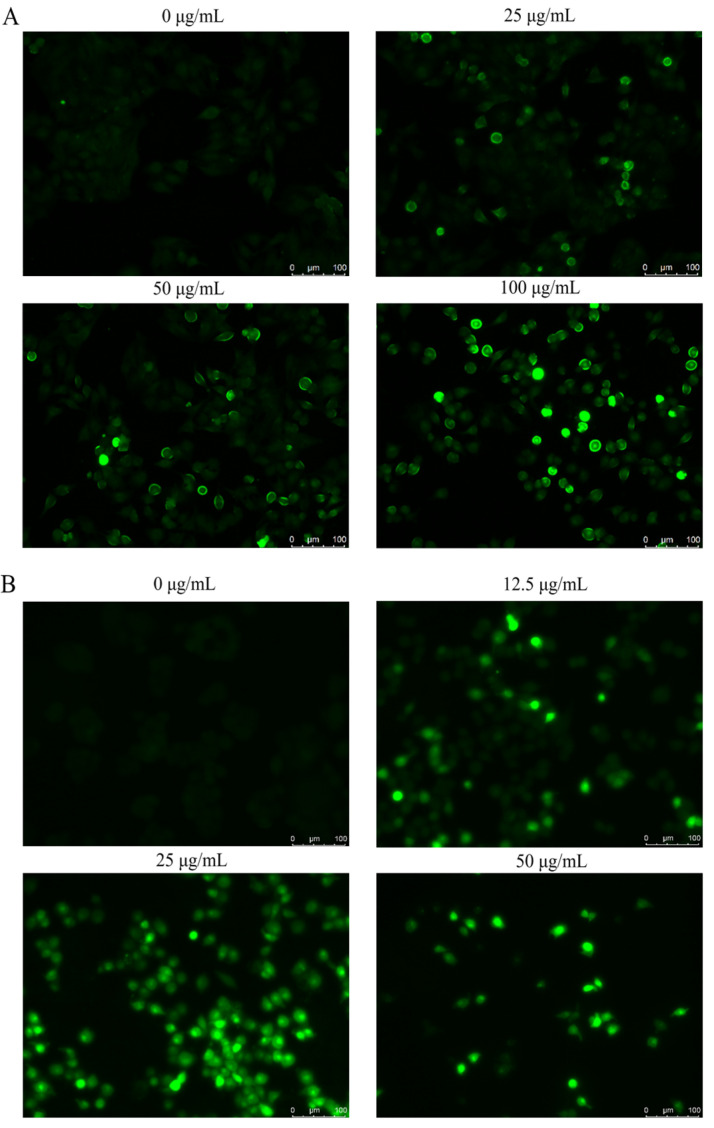
Intracellular ROS generation in HepG2 cells detected by DCFH-DA staining using fluorescence microscope after treatment with indicated concentrations of (**A**) *Ffsp-50* (0, 25, 50, and 100 μg/mL) and (**B**) *FTX* (0, 12.5, 25, and 50 μg/mL) for 24 h. Images are representative of three independent experiments (magnification ×200).

Intracellular ROS accumulation was determined by fluorescence analysis to assess the oxidative stress status of HepG2 cells. As shown in Figure 6A,B, treatment with increased concentrations of *Ffsp-50* or *FTX* caused upregulated intracellular fluorescence intensity along with a gradual decrease in cellular confluence and morphological changes including cell rounding, shrinkage, and increased intercellular space. Taken together, our result demonstrated that both *Ffsp-50* and *FTX* caused the overproduction of intracellular ROS and oxidative stress in HepG2 cells.

### 3.6. Phylogenetic Analysis

**Figure 7:**
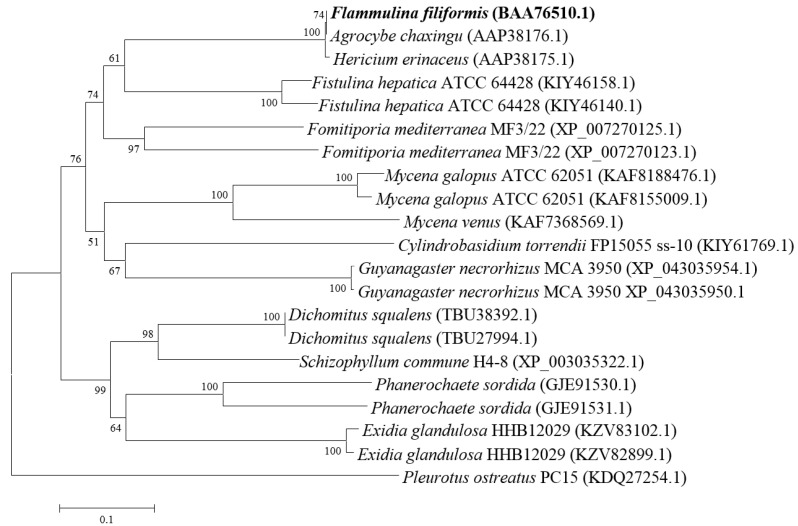
Phylogenetic tree of *FTX* protein in *F. filiformis* and other fungi.

Phylogenetic analysis was executed to evaluate the evolutionary relationship between *F. filiformis* and other fungi. As shown in Figure 7, *FTX* protein in *F. filiformis* (GenBank accession number: BAA76510.1) showed a high similarity with the homologue protein from *Agrocybe chaxingu* (GenBank accession number: AAP38176.1). Protein–protein BLAST (NCBI) revealed that its similarity value with *FTX* homologue protein in *Hericium erinaceus* (GenBank accession number: AAP38175.1), *Fistulina hepatica* ATCC 64428 (GenBank accession number: KIY46158.1), and *Fomitiporia mediterranea* MF3/22 (GenBank accession number: XP_007270125.1) was 99.63% (E value 0), 55.47% (E value 9 × 10^−94^), and 53.76% (E value 3 × 10^−97^), respectively. Phylogenetic analysis indicated that *FTX* protein was relatively conserved in basidiomycetes.

### 3.7. Analysis of Gene Expression Profile

qPCR was carried out to analyze the expression pattern of *FTX* during the growth and development of *F. filiformis.* As shown in Table 2, the relative expression level of *FTX* was significantly upregulated throughout the development of the fruiting body as compared with that in the mycelium (*p* < 0.05 or 0.01). It is worth noting that the relative expression level of *FTX* in the primordium and the pileus in the elongation and maturation stages was 1029.20 *±* 36.36 (*p* < 0.01), 1339.03 *±* 115.68 (*p* < 0.05), and 741.86 *±* 43.79 times (*p* < 0.01) of that in the mycelium, respectively.

## 4. Discussion

Edible mushrooms have become increasingly popular and consumed worldwide, especially owing to their good content of proteins. As an alternative to animal protein, edible mushrooms can be processed to obtain a wide spectrum of food products and may enhance their functional properties, endowing them with an added value as well [30,31]. Recently, *F. filiformis* cultivation has rapidly improved because of its high nutritional value and delicious taste, making it the fourth most popular edible mushroom worldwide. However, the generated root waste, despite having similar nutritional value to fruiting bodies, is often abandoned, thereby increasing treatment costs and bringing a huge burden to edible mushroom companies. Therefore, there is an urgent need to develop health and/or medicinal functions in the fruiting bodies to increase utilization and added value [32]. *F. filiformis* was recorded in the *Chinese Materia Medica* (*Zhong Hua Ben Cao*) as possessing liver-tonifying, stomach-benefiting, and antitumor properties [33]. To our best knowledge, little research regarding the antitumor activity of protein from *F. filiformis* exists. In this study, protein fractions were prepared from the fruiting body of *F. filiformis* by NaCl extraction and partitioned by gradual (NH_4_)_2_SO_4_ precipitation. They were systematically evaluated for growth-inhibitory effects against three GI cancer cell lines, viz. HT-29, SGC-7901, and HepG2. Among these protein fractions, *Ffsp-50,* dominated by *FTX* (Figure 1 and Table 1), was shown to be the most cytotoxic against cancer cells, as shown in Figure 4A–C. These findings prompted us to investigate whether *FTX* possessed the same or even stronger cytotoxic effects. As expected, *FTX* was purified and characterized with an *M_w_* of 26.78 kDa (Figure 2 and Figure 3), which significantly restrained cancer cell growth, especially for HepG2, with a time- and dose-dependent relationship (Figure 4D–F).

Cancer is a group of diseases characterized by uncontrolled cell growth and/or reduced cellular apoptosis, which is used as a valid target for anticancer therapy [34]. Recent studies have shown *FTX* could assemble into a ring-shaped oligomer to produce a hydrophilic pore of 4–5 nm in the cell membrane, thus resulting in potassium ion (K^+^) efflux from human erythrocytes and cell swelling [35,36]. Subsequent research has revealed that *FTX* exposure allows for a rapid calcium ion (Ca^2+^) influx from the extracellular part into Caco-2 cells, likely through membrane pore formation [28]. Compelling evidence has indicated that K^+^ efflux and Ca^2+^ influx are pivotal early steps in cell apoptosis [37,38,39,40]. Consequently, it was speculated that *FTX* possessed a cell apoptosis-inducing effect. As confirmed by DAPI staining and flow cytometry analysis, *Ffsp-50* and *FTX* triggered apparent apoptosis in HepG2, as evident in Figure 5. These findings demonstrated that the growth-inhibitory effects of *Ffsp-50* and *FTX* were at least partially attributable to their pro-apoptotic activities.

ROS act as essential signaling molecules in cancer. However, toxic levels of ROS generation in cancer are antitumorigenic, resulting in an increase in oxidative stress and induction of tumor cell apoptosis or death [41]. Mounting evidence has shown that conventional anticancer drugs exert chemotherapeutic effects by installing oxidative stress and ROS-mediated apoptosis in cancer cells [42]. A recent study showed that K^+^ efflux and Ca^2+^ influx elicited mitochondrial ROS generation and oxidative stress [43], eventually activating the mitochondria-dependent apoptosis pathway [44]. Our result demonstrated that both *Ffsp-50* and *FTX* caused an overproduction of intracellular ROS (Figure 6), probably resulting in mitochondria-dependent apoptosis in HepG2 cells. In addition, *FTX* was identified as a relatively conserved protein in basidiomycetes (Figure 7), indicating some conserved molecular mechanisms in multicellular complexity [45]. Gene expression profile analysis will help to understand its biofunctional role in the developmental process [46]. Recent transcriptome data revealed that *FTX* ranked the fifth in the top twenty upregulated genes induced in the primordium relative to the vegetative mycelium [47]. Gene expression analysis showed that *FTX* expression was particularly highly upregulated in the primordium as well as the pileus of the fruiting body during the elongation and maturation stages (Table 2). Therefore, the specific developmental stage and tissue upregulated expression of *FTX* implied its potential participation in the growth and development of the fruiting body.

Collectively, our study has shown that *FTX* could induce ROS generation and apoptosis in HepG2 cells. Its biofunctional role in the growth and development of *F. filiformis* warrants further investigation.

## 5. Conclusions

*F. filiformis* can be used as a functional food and has great potential for developing medical and health products. The results of our investigation provided insight into the antigastrointestinal cancer activity of *FTX*, which could serve as a biological source of health-promoting and biomedical applications. Furthermore, *FTX*, identified as a relatively conserved protein in basidiomycetes, was shown to be developmentally regulated. The biofunctional role of *FTX* in the development of *F. filiformis* emerged as an interesting scientific problem, thus meriting deeper exploration.

## Figures and Tables

**Table 1:** The protein composition of *Ffsp-50* determined by HPLC-MS/MS.

Gene	Mw (kDa)	%Con. (95%)	Peptides (95%)	Spectra	Conserved Domain
*Ffg414*	30.095	91.54	408	694	TEER-decreasing protein (TDP)
*Ffg11399*	212.929	81	471	603	Rhs repeat-associated core domain
*Ffg13516*	54.783	84.06	229	323	MAC/Perforin domain
*Ffg12369*	22.809	94.63	179	283	FDS protein
*Ffg13517*	51.052	86.90	117	173	MAC/Perforin domain
*Ffg13515*	52.516	93.19	114	129	MAC/Perforin domain

Note: Con. (95%): proportion of peptide segments in protein sequence with at least 95% confidence. Peptides (95%): number of peptide segments after removal of repeats with at least 95% confidence. Spectra: number of secondary mass spectra with corresponding protein. Conserved domain: structural and functional domain of protein predicted using the Conserved Domain Search service (CD-Search).

**Table 2:** Relative expression level of *FTX* in tissues of *F. filiformis* at different developmental stages.

Tissue Samples	Expression Level
Mycelium	1.00 ± 0.03
Primordium	1029.20 *±* 36.36 **
Young fruiting body	339.73 *±* 45.23 *
Stipe in elongation stage	332.65 *±* 31.67 *
Pileus in elongation stage	1339.03 *±* 115.68 *
Stipe in maturation stage	207.86 *±* 16.06 **
Pileus in maturation stage	741.86 *±* 43.79 **

Amplifications for each sample were performed with three technical replicates. Relative gene expression levels were calculated using the 2^−ΔΔCt^ method. * *p* < 0.05, ** *p* < 0.01, compared with relative expression level of *FTX* in mycelium.

## Data Availability

Data are contained within the article.
